# Rezidivierende Hämolysen und Eisenüberladung unklarer Genese

**DOI:** 10.1007/s00108-024-01801-4

**Published:** 2024-10-01

**Authors:** Laura Distelmaier, Christian Gebhard, Antje Holzäpfel, Michael von Bergwelt-Baildon, Sebastian Theurich, Holger Cario, Karsten Spiekermann

**Affiliations:** 1 Medizinische Klinik und Poliklinik 3 für Hämatologie und Onkologie, Tagesklinik Campus Innenstadt, Ludwig-Maximilians-Universität München, Ziemssenstr. 1, 80336 München, Deutschland; 2 Medizinisch Genetisches Zentrum München, München, Deutschland; 3 Bayerisches Zentrum für Krebsforschung (BZKF), LMU Universitätsklinikum, München, Deutschland; 4 Standort München und Deutsches Krebsforschungszentrum Heidelberg, Deutsches Konsortium für Translationale Krebsforschung (DKTK), Heidelberg, Deutschland; 5 Klinik für Kinder- und Jugendmedizin, Universitätsklinikum Ulm, Ulm, Deutschland; 6 Zentrum f. Seltene Störungen der Hämatopoese und Immundefekte (ZSHI Ulm), Universitätsklinikum Ulm, Ulm, Deutschland

**Keywords:** Xerozytose, Erythrozytenmembrandefekt, Osmotische Resistenz, Gendiagnostik, Morbus Meulengracht, Xerocytosis, Erythrocyte membrane defect, Osmotic resistance, Genetic testing, Gilbert disease

## Abstract

Vorgestellt wird der Fall eines 33-jährigen Mannes mit rezidivierenden Ikterusepisoden und Hämolysen seit der Kindheit, die lange als Morbus Meulengracht fehlgedeutet wurden. In der Folge entwickelten sich bei dem Patienten auch eine Splenomegalie und Gallensteine in Verbindung mit einer Eisenüberladung. Auf Basis gendiagnostischer Untersuchungen wurde eine hereditäre Xerozytose diagnostiziert, das heißt eine Erythrozytenmembranstörung, die rezidivierende Hämolysen verursacht. Die Diagnose der Xerozytose ist oft eine Herausforderung. Die Häufigkeit der Erkrankung wird möglicherweise unterschätzt, da eine mikroskopische Blutuntersuchung häufig keine typischen Befunde liefert. Zudem fällt der Eosin-5-Maleimid(EMA)-Test, der zur Diagnose anderer Erythrozytenmembranstörungen eingesetzt wird, normal aus. In Fällen von Splenomegalie, Eisenüberladung und rezidivierender Hämolyse oder im Falle eines klinisch diagnostizierten Morbus Meulengracht in Kombination mit einem der zuvor genannten Symptome sollten weiterführende Untersuchungen und gegebenenfalls auch eine gendiagnostische Abklärung erwogen werden.

## Abstract (en)

The case of a 33-year-old male with recurrent icterus and hemolysis since childhood that was long mistaken for Gilbert disease is presented. Subsequently, the patient also developed splenomegaly and gallstones together with iron overload. Genetic testing revealed the diagnosis of hereditary xerocytosis, which is an erythrocyte membrane disorder causing recurrent hemolysis. Xerocytosis is often challenging to diagnose and the frequency of the condition might be underestimated as there are often no typical findings in the microscopic differential blood count, and Eosin-5-maleimide dye (EMA) test, which is used to diagnose other erythrocyte membrane disorders, is normal. In cases of splenomegaly, iron overload and recurrent hemolysis, or in the case of a clinical diagnosis of Gilbert disease together with one of the above-mentioned symptoms, further investigations and possibly also genetic testing should be considered.

## Anamnese

Ein 33-jähriger Mann stellte sich zur Abklärung von rezidivierenden Hämolysen und erhöhten Eisenspeicherparametern in unserer Ambulanz vor.

Bei dem Patienten war seit der Kindheit wiederholt ein Ikterus aufgetreten, zudem zeigten sich im Blut rezidivierend Hämolysezeichen und ein erhöhter Ferritinwert. Eine grenzwertige Splenomegalie war im Verlauf zudem festgestellt worden. Aufgrund einer symptomatischen Cholezystolithiasis erfolgte im Alter von 20 Jahren eine Cholezystektomie.

In der Familie litten mehrere Familienmitglieder mütterlicherseits (Mutter, Onkel sowie Großvater und Urgroßmutter) unter rezidivierenden Ikterusepisoden.

Bei dem Patienten waren in der Kindheit klinisch ein Morbus Meulengracht sowie ein Glukose-6-Phosphat-Dehydrogenase(G6PDH)-Mangel vermutet worden. Vor einigen Jahren waren eine umfangreiche Abklärung mittels mikroskopischer Untersuchung des Blutbilds, Hämoglobinanalyse und G6PDH-Aktivitätsmessung sowie eine Sphärozytosediagnostik mittels Eosin-5-Maleimid(EMA)-Test und „acidified glycerol lysis test“ (AGLT) erfolgt. Alle Untersuchungen hatten, ebenso wie eine genetische Untersuchung bezüglich eines Morbus Meulengracht, keinen Anhalt für die Ursache der Symptomatik ergeben.

## Befund

**Abb. 1:**
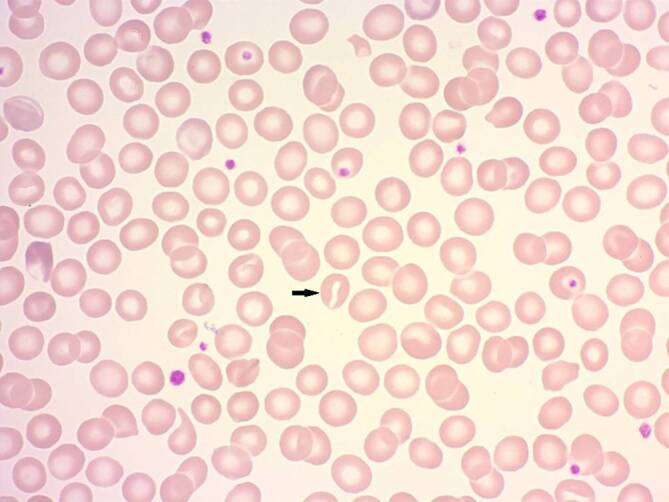
Blutausstrich mit einzelnen Stomatozyten (siehe *Pfeil*)

Laborchemisch lagen hier nur milde Hämolysezeichen, ein mit 13,6 g/dl normaler Hämoglobinwert, ein normales MCV, eine Erhöhung des indirekten Bilirubins und eine Eisenüberladung mit einem Ferritin von 715 ng/ml vor. Außerdem bestand eine Splenomegalie mit einem Längsdurchmesser von 14 cm. Der periphere Blutausstrich (Abb. 1) zeigte vereinzelte Stomatozyten, aber keinen wegweisenden Befund.

Zur weiteren Abklärung der Befunde erfolgte eine genetische Paneldiagnostik, welche zum einen eine heterozygote Variante HFE: p.C282Y im HFE-Gen zeigte, welche jedoch ohne zusätzliche prädisponierende Faktoren nicht zu einer relevanten Eisenüberladung führt. Zudem wurde aber auch die pathogene Variante NM_002250.3:c.1055G > A (p.Arg352His) im Potassium-calcium-activated-channel-subfamily-N-member-4-Gen *(KCNN4)* in heterozygotem Zustand festgestellt.

## Diagnose

Bei dem Patienten konnte eine dehydrierte hereditäre Stomatozytose Typ 2 (DHS2, OMIM #616689) diagnostiziert werden. Dabei handelt es sich um eine Form der auch als hereditäre Xerozytosen bekannten Erythrozytenmembrandefekte, die zu einer chronischen Hämolyse und Eisenüberladung führen. Die vorliegende Variante im KCNN4-Gen wurde erstmals 2015 beschrieben [1] und wird nach ACMG-Klassifikation als „pathogen“ klassifiziert.

Der Patient berichtete uns im Verlauf, dass genetische Untersuchungen das Vorliegen dieser Variante, passend zu deren klinischer Präsentation mit rezidivierendem Ikterus, auch bei der Mutter und dem Onkel des Patienten bestätigen konnten.

## Therapie und Verlauf

Dem Patienten wurden zunächst Kontrolluntersuchungen in 4‑ bis 6‑monatigen Abständen empfohlen. Bei Anstieg des Ferritinwerts über 1000 ng/ml sollte eine Lebereisenbestimmung erfolgen. Im Fall einer manifesten Eisenüberladung wäre angesichts des guten Hämoglobingehalts eine Therapie mit Aderlässen zu erwägen. Sollten diese vom Patienten nicht toleriert werden, wäre alternativ eine vorübergehende medikamentöse Eiseneliminationstherapie möglich. Eine Splenektomie wird auch in Fällen mit einer stärker ausgeprägten Anämie nicht empfohlen, da zum einen der Einfluss auf die Symptomatik gering ist und sich in Studien zudem ein deutlich erhöhtes Thromboserisiko von Xerozytose-Patienten nach Splenektomie gezeigt hat [2].

## Diskussion

Bei den Xerozytosen oder dehydrierten Stomatozytosen handelt es sich um seltene Anämien, bei denen es zu einer gestörten Membranpermeabilität kommt. Genetisch liegen bei den Xerozytosen *Gain-of-function*-Mutationen in den Genen *PIEZO1* (DHS1, OMIM #194380) oder, wie bei unserem Patienten, *KCNN4* (DHS2, OMIM #616689) vor. Der Phänotyp kann sehr unterschiedlich ausgeprägt sein. Meist liegt eine gut kompensierte chronische Hämolyse mit einem milden Ikterus vor [3]. Typische Veränderungen in Form von Xerozyten oder Stomatozyten im peripheren Blutausstrich fehlen häufig. Aus diesem Grund wird die Diagnose oft, wie im vorliegenden Fall, jahrelang übersehen und die Häufigkeit dieser Erkrankung wahrscheinlich unterschätzt. Mögliche Anzeichen einer Xerozytose können eine unerklärte, oft ausgeprägte Eisenüberladung in Verbindung mit einer Splenomegalie, auffälligen Hämolyseparametern und rezidivierenden hämolytischen Krisen sein. Auch der rein klinische Verdacht auf einen Morbus Meulengracht in Kombination mit einem der oben genannten Symptome sollte weiter abgeklärt werden. Typischerweise zeigt sich im Labor ein normales Hämoglobin oder eine milde Anämie kombiniert mit einem normalen MCV oder milder Makrozytose, die MCHC ist oft erhöht. Laborchemisch kann eine Pseudohyperkaliämie vorliegen. In seltenen Fällen gibt die ergänzende mikroskopische Diagnostik des Blutbilds dann erste Hinweise. Im EMA-Test, welcher bei anderen Membrandefekten wie der Sphärozytose typische pathologische Befunde ergibt, zeigt sich im Falle der Xerozytose ein normaler Befund. Im AGLT zeigt sich eine eher erhöhte osmotische Resistenz, welche wegweisend sein kann. Charakteristisch ist eine in Relation zum Ausmaß der Anämie und der Hämolyse eher ungewöhnlich ausgeprägte Hyperferritinämie, die über die bei Patienten mit chronischer Hämolyse im Laufe des Lebens zu erwartende Eisenüberladung hinausgeht. Dafür wird bei Patienten mit Xerozytose aufgrund einer *PIEZO1*-Variante (DHS1) deren hemmender Einfluss auf die Synthese von Hepcidin, dem Hauptregulator des Eisenstoffwechsels, verantwortlich gemacht. Vermittelt wird dies über Veränderungen der hepatozellulären Kalziumkonzentration [4]. Für *KCNN4* gibt es bisher solche experimentellen Daten nicht.

Bei klinischem und laborchemischem Verdacht wird die Diagnose einer DHS in der Regel über eine genetische Untersuchung gesichert. Für die DHS1, nicht aber für die bei unserem Patienten vorliegende DHS2 ist eine Diagnosestellung prinzipiell auch über eine funktionelle Untersuchung mittels Ektazytometrie möglich, die aber nur sehr eingeschränkt zur Verfügung steht.

## Fazit für die Praxis

- Aufgrund der Seltenheit und der erschwerten Diagnostik wird die Xerozytose leicht übersehen.
- Oft sind nur milde Hämolysezeichen, zusammen mit einem erhöhten Ferritinwert, feststellbar. Blutausstrich und EMA-Test sind, anders als z. B. bei der Sphärozytose, oft nicht wegweisend. Die osmotische Resistenz (bei Sphärozytose typischerweise erniedrigt) ist hingegen oft auffällig erhöht.
- Bei Patienten, bei denen auffällige Symptome wie rezidivierende Hämolyse, Ikterus, Splenomegalie oder erhöhte Eisenparameter vorliegen, sollte auch an seltenere Formen von Erythrozytenmembrandefekten gedacht werden.
